# Transdifferentiation of α-1,3-galactosyltransferase knockout pig bone marrow derived mesenchymal stem cells into pancreatic *β*-like cells by microenvironment modulation

**DOI:** 10.5713/ajas.19.0796

**Published:** 2020-02-25

**Authors:** Imran Ullah, Ran Lee, Keon Bong Oh, Seongsoo Hwang, Youngim Kim, Tai-Young Hur, Sun A Ock

**Affiliations:** 1 Animal Biotechnology Division, National Institute of Animal Science, Rural Development Administration, Wanju 55365, Korea; 2 Department of Biochemistry, Faculty of Biological Sciences, Quaid-i-Azam University, Islamabad 45320, Pakistan

**Keywords:** N2B27, GalTKO BM-MSCs, Epigenetic Modifications, Cytidine, Pancreatic β-like Cells

## Abstract

**Objective:**

To evaluate the pancreatic differentiation potential of α-1,3-galactosyltransferase knockout (GalTKO) pig-derived bone marrow-derived mesenchymal stem cells (BM-MSCs) using epigenetic modifiers with different pancreatic induction media.

**Methods:**

The BM-MSCs have been differentiated into pancreatic β-like cells by inducing the overexpression of key transcription regulatory factors or by exposure to specific soluble inducers/small molecules. In this study, we evaluated the pancreatic differentiation of GalTKO pig-derived BM-MSCs using epigenetic modifiers, 5-azacytidine (5-Aza) and valproic acid (VPA), and two types of pancreatic induction media – advanced Dulbecco’s modified Eagle’s medium (ADMEM)-based and N2B27-based media. GalTKO BM-MSCs were treated with pancreatic induction media and the expression of pancreas-islets-specific markers was evaluated by real-time quantitative polymerase chain reaction, Western blotting, and immunofluorescence. Morphological changes and changes in the 5′-C-phosphate-G-3′ (CpG) island methylation patterns were also evaluated.

**Results:**

The expression of the pluripotent marker (POU class 5 homeobox 1 [OCT4]) was upregulated upon exposure to 5-Aza and/or VPA. GalTKO BM-MSCs showed increased expression of neurogenic differentiation 1 in the ADMEM-based (5-Aza) media, while the expression of NK6 homeobox 1 was elevated in cells induced with the N2B27-based (5-Aza) media. Moreover, the morphological transition and formation of islets-like cellular clusters were also prominent in the cells induced with the N2B27-based media with 5-Aza. The higher insulin expression revealed the augmented trans-differentiation ability of GalTKO BM-MSCs into pancreatic β-like cells in the N2B27-based media than in the ADMEM-based media.

**Conclusion:**

5-Aza treated GalTKO BM-MSCs showed an enhanced demethylation pattern in the second CpG island of the OCT4 promoter region compared to that in the GalTKO BM-MSCs. The exposure of GalTKO pig-derived BM-MSCs to the N2B27-based microenvironment can significantly enhance their trans-differentiation ability into pancreatic β-like cells.

## INTRODUCTION

Diabetes mellitus (DM) is a hyperglycemic condition induced either by a failure to secrete insulin due to dysfunctional *β*-cells (type I) or insulin resistance (type II). According to the International Diabetes Federation, in 2017, an estimated 425 million adults were diagnosed with DM worldwide and by 2040 this number is expected to be over 600 million. Patients with diabetes fail to maintain normal plasma glucose levels [1,2], which could lead to severe physiological complications. Insulin therapy together with dietary regulations and regular exercise is the only treatment option for the millions of people suffering from DM. However, this approach is not a permanent treatment and is a temporary control strategy that does not cure DM permanently. Transplantation of *β*-cells is a strategy by which the dysfunctional *β*-cells are replaced with healthy ones to enable the permanent maintenance of normal plasma glucose levels [3]. Although allogeneic transplantation of cadaveric islets is feasible, the limited availability of islets and the need for continuous immunosuppression in the recipients debilitate its clinical success [4]. Therefore, in the present era of modern translational medicine, researchers are trying to overcome this limitation by generating effective *β*-cells using stem cell technologies. Previously, efforts have been made to generate pancreatic *β*-like cells from various kinds of stem cells including embryonic stem cells [5,6], human fibroblasts [7], induced pluripotent cells (iPSCs), and human bone marrow mesenchymal stem cells (BM-MSCs) [8,9]. Among the adult stem cells, the BM-MSCs have a higher proliferation capacity and can retain this capacity for longer periods and multiple passages [10]. The BM-MSCs have been used for the generation of pancreatic *β*-like cells either by inducing the overexpression of key transcription regulatory factors [11] or by the addition of specific soluble inducers/small molecules in the cell culture medium [11,12]. The effectiveness and extent of stem cell differentiation are modulated by the external microenvironment and internal genetic programming. Previous studies have suggested that the microenvironment plays a crucial role in the differentiation and survival of stem cells. This has prompted efforts to modulate the microenvironment to mimic various stages of pancreatic development for successful differentiation of BM-MSCs into pancreatic *β*-like cells. Epigenetic mechanisms either in the form of DNA methylations or histone modifications play important roles in cell-fate determination during pancreatic development [13]. Different epigenetic modification factors like histone deacetylases inhibitors (Trichostatin A, valproic acid [VPA] and TMP269) and DNA demethylases (5-azacytidine [5-Aza]) were successfully used to differentiate pluripotent stem cells into pancreatic *β*-like cells [7,14,15].

Another strategy for the development of pancreatic *β*-like cells is the use of porcine stem cells. This is hindered by the immune rejection response due to species differences [16]. The main immunological barrier in pig-to-human transplantation is the binding of human natural anti-galactose (Gal) antibody to the galactose-alpha 1,3-galactose epitopes richly expressed in pig cells. This effect has been scaled down by generating α-1,3-galactosyltransferase knockout (GalTKO) transgenic pigs [17].

We aimed at enhancing the differentiation ability of porcine GalTKO BM-MSCs into pancreatic *β*-like cells by mimicking the cellular microenvironment using small molecules and epigenetic modulating factors such as 5-Aza and VPA. Additionally, we investigated whether the epigenetic factors or the cellular microenvironment is more important for the induction of GalTKO BM-MSCs into pancreatic *β*-like cells. To our knowledge, this is the first attempt of generation of pancreatic *β*-like cells derived from GalTKO pig-derived BM-MSCs.

## MATERIALS AND METHODS

### Reagents and media

Unless otherwise specified, all chemicals were purchased from Sigma-Aldrich Corporation. (St. Louis, MO, USA), and the cell culture media were obtained from Gibco (Life Technologies, Carlsbad, CA, USA).

### Animal experiment

All animal experiments were carried out in accordance with the Guide for the Care and Use of Laboratory Animals of our institution and were approved by the Animal Ethics Committee of the National Institute of Animal Science, Rural Development Association, Republic of Korea (NIAS2015-720).

### Isolation of BM-MSCs from GalTKO pig

BM-MSCs were isolated as described previously [18]. After isolation, GalTKO BM-MSCs were cultured in advanced Dulbecco’s modified Eagle’s media (ADMEM) supplemented with 10% fetal bovine serum (FBS), 1X GlutaMAX (Gibco, USA), and 1% penicillin-streptomycin (10,000 IU and 10,000 μg/mL) at 37°C and 5% CO_2_ in a humidified incubator. After reaching confluence, cells were sub-cultured for further analyses.

### Induction of BM-MSCs with epigenetic factors

GalTKO BM-MSCs were treated with optimized concentrations of epigenetic factors as previously reported [19,20]. Briefly, GalTKO BM-MSCs were seeded at a density of 1×10^5^ cells/35 mm dish in ADMEM supplemented with 10% FBS for 24 hours (day 0), followed by treatment with 1 μM 5-Aza and/or 50 μg/mL VPA and were cultured at 37°C and 5% CO_2_ in a humidified incubator for next 24 hours (day 1). Based on 5-Aza and/or VPA pre-treatment, the BM-MSCs were divided into four pancreatic induction groups – Untreated, 5-Aza group, VPA group, and the 5-Aza/VPA group. Naive GalTKO BM-MSCs constituted the control group.

### BM-MSCs differentiation into pancreatic *β*-like cells with the pancreatic conditioned microenvironment

Pancreatic induction was performed as described previously with slight modifications [9]. Briefly, after one day of 5-Aza and/or VPA treatment (day 2), the culture media was replaced with an ADMEM-based pancreatic induction media (ADMEM-based media) containing 0.5 mmol/L β-mercaptoethanol and incubated for more 2 days. The medium was again replaced with ADMEM containing 1% nonessential amino acids, 20 ng/mL basic fibroblast growth factor, 20 ng/mL epidermal growth factor, 2% B27 supplement, and 2 mM/L L-glutamine and incubated for eight days. Finally, the induced GalTKO BM-MSCs were cultured into ADMEM containing 10 ng/mL betacellulin, 10 ng/mL activin A, 2% B27 supplement, and 10 mM/L nicotinamide (STEMCELL Technologies Inc., Vancouver, BC, Canada) until one month (~ day 30). After complete induction, the cells were subjected to characterization.

In addition, we used an N2B27-based pancreatic induction media (N2B27-based media) with minor modifications [7]. After 1 day of 5-Aza and/or VPA treatment (day 2), the GalTKO BM-MSCs were cultured in N2B27 basal medium (DMEM/F12 medium supplemented with N2, B27, 0.1 mM *β*-mercaptoethanol, 2 mM glutamine, 1 mM MEM nonessential amino acids, 0.5% bovine serum albumin [BSA] and 100 ng/mL of basic fibroblast growth factor) including 30 ng/mL activin A for 6 days. Cells were further treated with N2B27 basal medium including 10 M retinoic acid for two days. Two days later, the medium was refreshed and replaced with N2B27 basal medium supplemented with 1% B27, 20 ng/mL basic fibroblast growth factor, and 1% insulin-transferrin-selenium (STEMCELL Technologies lnc., Canada) to enhance the differentiation potential until two months. Media was replaced after every three days, until maturation. After complete induction, the cells were subjected to characterization.

### Real-time quantitative polymerase chain reaction analysis

Total RNA was isolated from GalTKO BM-MSCs and pancreatic *β*-like cells differentiated for one month using the RNeasy mini kit (Qiagen GmbH, Hilden, Germany) and quantified with a spectrophotometer (NanoDrop 1000, Thermo Scientific, Wilimington, DE, USA). Complementary DNA (cDNA) was synthesized from total purified RNA (1 μg) using the Omniscript reverse transcription kit (Qiagen, Germany) with 10× RT buffer, dNTP mix, RNase inhibitor, 10 μM OligodT primer at 37°C for 1 h. real-time quantitative polymerase chain reaction (RT-qPCR) reaction was performed using StepOnePlus Real-Time PCR System (Foster City, CA, USA) with SYBR Green master mix (Thermo Fisher Scientific, Carlsbad, CA, USA) supplemented with a 10 μM specific primer set (Table 1). All experiments were carried out in five replicates and glyceraldehyde-3-phosphate dehydrogenase (*GAPDH*) was used as an internal control. PCR products were electrophoresed and the amplification of POU class 5 homeobox 1 (*OCT4*), SRY (sex determining region Y)-box 2 (*SOX2*), Nanog homeobox (*NANOG*), and *GAPDH* were evaluated.

### Immunofluorescence analysis of pancreatic islets-specific proteins

For immunofluorescence analysis, same numbers of cells were seeded on the cover glass in micro-well plates and were induced with differentiation media for one month. After differentiation, the cells were fixed with 4% formaldehyde and permeabilized with 0.1% Triton X-100 supplemented with 5% BSA. The cells were blocked with 5% BSA for 1 h and then incubated with the primary antibodies – “rabbit Anti-Glucagon (1:500) (Millipore, Temecula, CA, USA), rabbit Anti-somatostatin (1:500) (Millipore, USA), mouse Anti-pro insulin c-peptide (1:200) (Millipore, USA), and rabbit Anti-pancreatic polypeptide (PP; 1:1,000) (Millipore, USA)”, at 4°C overnight. Cells were then washed three times with Dulbecco’s phosphate buffer saline and incubated with goat anti-rabbit (Thermo Fisher Scientific, Rockford, IL, USA) and Goat anti-mouse (Thermo Fisher Scientific, USA) antibodies at 37°C for 1 h. For nuclear staining, cells were treated with 1 μg/mL 4′,6-diamidino-2-phenylindole for 30 min at room temperature (RT). Finally, the cells were mounted with Vectashield Antifade Mounting Medium (Vector Laboratories, Burlingame, CA, USA) and observed under a fluorescence microscope (Leica DMI 6000B, Wetzlar, Germany).

### Western blot analysis

Protein lysates were prepared from pancreatic *β*-like cells induced with the N2B27-based media for two months using radioimmunoprecipitation assay buffer (Pierce, Rockford, IL, USA) containing protease inhibitor and were quantified using a BCA protein assay kit (Pierce, USA). The protein samples (7 μg) were resolved by 8% to 12% sodium dodecyl sulfate-polyacrylamide gel electrophoresis for 3 h at 100 V and blotted on to a polyvinylidene difluoride membrane (Bio-Rad, Hercules, CA, USA) overnight at 30 V. The membranes were then blocked with 5% BSA in Tris-buffered saline (1× TBS) for 1 h at RT, followed by washing in 0.1% Tris-buffered saline-Tween (TBST). The membranes were incubated with primary antibodies, rabbit anti-PDX1 (Abcam, Cambridge, MA, USA; 31 kDa) and mouse anti-*β*-actin (42 kDa), overnight at 4°C. After washing three times with 0.1% TBST, the membranes were incubated with horseradish peroxidase-conjugated donkey anti-rabbit (Abcam, USA) and goat anti-mouse (Abcam, USA) secondary antibodies for 1 h at RT. Immunoreactivity was detected by enhanced chemiluminescence (ECL; SuperSignal West Pico chemiluminescent substrate, Pierce, USA). The membranes were then exposed to X-ray films in the dark for 10 s.

### Methylation pattern of the OCT4 5′-C-phosphate-G-3′ Island by bisulfite genomic sequencing

Genomic DNA was extracted from ear fibroblasts (EFs), GalTKO BM-MSCs and 5-Aza treated GalTKO BM-MSCs using DNeasy Blood & Tissue Kit (Qiagen, Germany) The DNA bisulfite reaction was carried out using the EpiTect Bisulfite kit “(Qiagen, Germany)”. Briefly, total DNA concentration of all samples was kept at 1 μg and the total volume was made up to 20 μL using RNase free water, after which 85 μL bisulfite mix and 35 μL DNA protect buffer was added to the sample. After the color of the bisulfite mix changed from green to blue, the samples were placed in a thermal cycler set at 95°C for 5 min (denaturation), followed by 60°C for 85 min (incubation), 95°C for 5 min (denaturation), 60°C for 175 min (incubation), and a finally held at 20°C. 5′-C-phosphate-G-3′ (CpG) island prediction including 2,000 bps nucleotides upstream and 500 bp downstream of the target sequence (*OCT4*; Gene ID: 5460) was performed by EMBOSS CpGplot program as previously described [21]. Three CpG islands in the promoter region of *OCT4* were identified and were used for the analyses. For sequencing analysis, the amplified *OCT4* CpG islands were cloned into a TA-cloning vector and 15 clones for each amplified fragment derived from individual alleles were sequenced. The individual CpGs are presented as methylated (●), un-methylated (○), or unknown (-).

### Statistical analyses

Data were analyzed by one-way analysis of variance using IBM SPSS statistics 24. PCR data are expressed as relative quantity (RQ) and error bars denote RQ±Min and RQ±Max. Differences were considered significant at p<0.05.

## RESULTS

### Epigenetic factors enhanced the stemness of BM-MSCs

**Figure 1:**
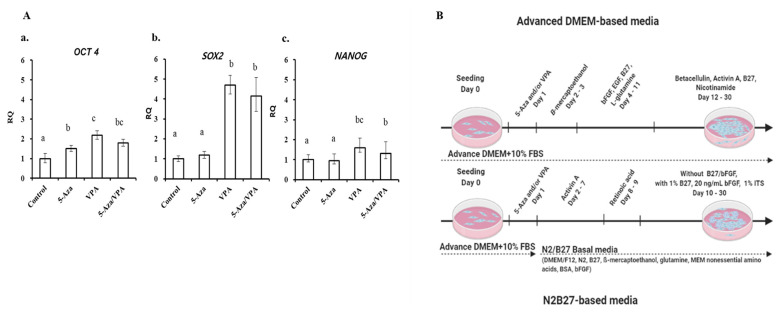
Real-time quantitative polymerase chain reaction analysis of pluripotent markers. (Aa) *OCT4*, (Ab) *SOX2*, and (Ac) *NANOG* in 5-azacytidine (5-Aza; 1 μM), valproic acid (VPA; 50 μg/mL), and 5-Aza/VPA groups as compared to control group (naïve GalTKO BM). (B) Schematic representation of GalTKO BM-MSCs differentiation into pancreatic *β*-like cells with ADMEM based- or N2B27 based-media. *OCT4*, POU class 5 homeobox 1; *SOX2*, SRY (sex determining region Y)-box 2; *NANOG*, Nanog homeobox; GalTKO, α-1,3-galactosyltransferase knockout; BM-MSCs, bone marrow-derived mesenchymal stem cells; ADMEM, advanced Dulbecco’s modified Eagle’s medium.

To evaluate the pluripotency of GalTKO BM-MSCs, cells from passage 3 were analyzed for the expression of pluripotent markers (*OCT4*, *SOX2*, and *NANOG*) using RT-qPCR. The expression of the pluripotent markers was upregulated upon treatment with 5-Aza/VPA. OCT4 expression of GalTKO BM-MSCs significantly increased with 5-Aza/VPA treatment as compared to the non-treated group (Figure 1Aa). Furthermore, *SOX2* and *NANOG* showed higher expression in the VPA-treated GalTKO BM-MSCs compared with other groups (Figure 1Ab, 1Ac). Interestingly, the GalTKO BM-MSCs treated with 5-Aza/VPA showed reduced expression of all three transcription factors compared to the VPA-treated group (Figure 1A).

### Expression of pancreatic islets-specific genes in GalTKO BM-MSCs induced using an ADMEM-based pancreatic induction media

**Figure 2:**
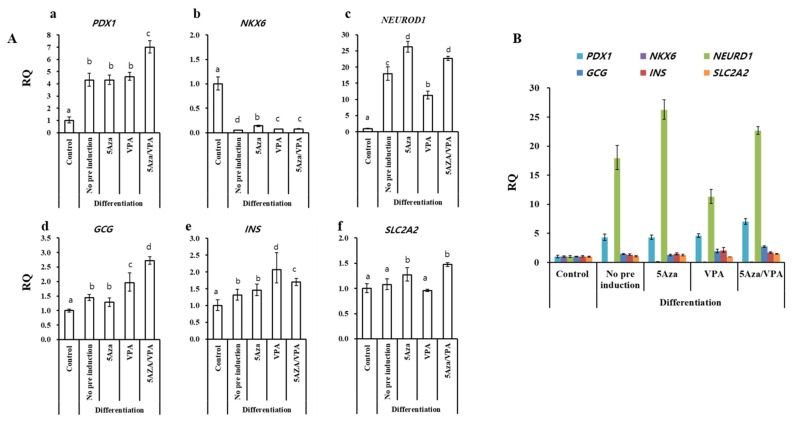
Real-time quantitative polymerase chain reaction (qPCR) analysis of pancreatic islets-specific markers after one month of cell differentiation by ADMEM-based media. (Aa) *PDX1*, (Ab) *NKX6*, (Ac) *NEUROD1*, (Ad) *GCG*, (Ae) *INS*, and (Af) *SLC2A2* expression in cells induced with the ADMEM-based induction media with 5-azacytidine (5-Aza; 1 μM), valproic acid (VPA; 50 μg/mL), and 5-Aza/VPA groups as compared to the control group (naïve GalTKO BM-MSCs) and no pre-induction group (differentiated pancreatic beta-like cells without prior 5-aza and/or VPA treatment). (B) Comparison of pancreatic markers expression among the groups. Letters a, b, and c indicate significant differences (p<0.05) in the expression of mRNA between different groups. *GAPDH* was used as an internal control. ADMEM, advanced Dulbecco’s modified Eagle’s medium; *PDX1*, pancreatic and duodenal homeobox 1; *NKX6*, NK6 homeobox 1; *NEUROD1*, neurogenic differentiation 1; *GCG*, glucagon; *INS*, insulin; *SLC2A2*, solute carrier family 2 (facilitated glucose transporter), member 2; GalTKO, α-1,3-galactosyltransferase knockout; BM-MSCs, bone marrow-derived mesenchymal stem cells; *GAPDH*, glyceraldehyde-3-phosphate dehydrogenase.

After treating the GalTKO BM-MSCs with the ADMEM-based media for one month (Figure 1B), the expression of pancreas-islets-specific markers: pancreatic and duodenal homeobox 1 (*PDX1*), NK6 homeobox (*NKX6*), neurogenic differentiation 1 (*NEUROD1*), glucagon (*GCG*), insulin (*INS*), and solute carrier family 2 (facilitated glucose transporter), member 2 (*SLC2A2*) in the newly generated pancreatic β-like cells were analyzed by RT-qPCR. All groups showed a significant increase in the expression of *PDX1*, *NEUROD1*, *GCG*, and *INS*, while *NKX6* showed a decrease in expression compared with that in the control (Figure 2A). Furthermore, among the pancreatic β-like cells, *NEUROD1* and *SLC2A2* in the 5-Aza group (Figure 2Ac, 2Af); *GCG* and *INS* in the VPA group (Figure 2Ad, 2Ae); and *PDX1*, *NEUROD1*, *GCG*, *INS*, and *SLC2A2* in the 5-Aza/VPA group (Figure 2Aa, 2Ac, 2Ad, 2Ae, 2Af) showed significantly higher expression as compared to the untreated group. The expression of pancreatic markers in the induced *β*-like cells confirmed their cell-fate transition from stem cells to *β*-like cells. Induction with the ADMEM-based media did not demonstrate a dramatic increase in the expression of pancreatic *β*-cells markers, in particular, extremely low expression of *INS* (1 to 2 fold) was observed; although *NEUROD1* showed a 25-fold increase in expression in the 5-Aza group as compared to the untreated group.

### Expression of pancreatic islets-specific genes in GalTKO BM-MSCs induced using an N2B27-based pancreatic induction media

**Figure 3:**
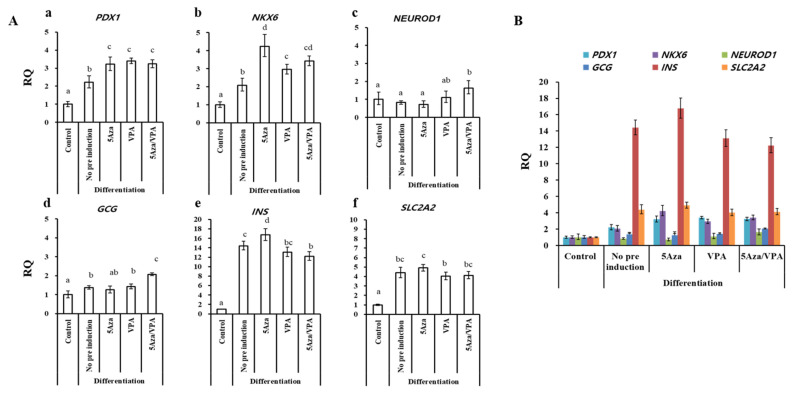
Real-time quantitative polymerase chain reaction analysis of pancreatic islets-specific markers after 1 month of cell differentiation by N2B27-based media. (Aa) *PDX1*, (Ab) *NKX6*, (Ac) *NEUROD1*, (Ad) *GCG*, (Ae) *INS*, and (Af) *SLC2A2* in cells induced with the N2B27-based induction media with 5-azacytidine (5-Aza; 1 μM), valproic acid (VPA; 50 μg/mL), and 5-Aza/VPA groups as compared to the control group (naïve GalTKO BM-MSCs) and no pre-induction group (differentiated pancreatic beta-like cells without prior 5-aza and/or VPA treatment). (B) Comparison of expression of pancreatic markers among the groups. Letters a, b, and c indicate significant differences (p<0.05) in the expression of mRNA between different groups. *GAPDH* was used as an internal control. *PDX1*, pancreatic and duodenal homeobox 1; *NKX6*, NK6 homeobox 1; *NEUROD1*, neurogenic differentiation 1; *GCG*, glucagon; *INS*, insulin; *SLC2A2*, solute carrier family 2 (facilitated glucose transporter), member 2; GalTKO, α-1,3-galactosyltransferase knockout; BM-MSCs, bone marrow-derived mesenchymal stem cells; *GAPDH*, glyceraldehyde-3-phosphate dehydrogenase.

GalTKO BM-MSCs induced with the N2B27-based media for one month (Figure 1B) were analyzed similarly to the GalTKO BM-MSCs induced with the ADMEM-based media. However, the expression pattern of pancreatic islets-specific markers was relatively different in the N2B27-based media induced cells compared to the ADMEM-based media induced cells (Figure 3A, B). The major difference was a significantly higher expression of *NKX6* (4-fold) (Figure 3Ab), *INS* (16.7 fold) (Figure 3Ae), and *SLC2A2* (4.9 fold) (Figure 3Af) in the 5-Aza group compared to that in the other groups. Furthermore, there was no significant difference in the expression of *PDX1* among the 5-Aza, VPA, and 5-Aza/VPA groups, while it showed an increase in expression in the 5-Aza/VPA+ ADMEM-based media group (Figure 2Aa, 3Aa). The expression of *NEUROD1* was downregulated while *NKX6* was upregulated in the cells induced with the N2B27-based media as compared with that in the cell induced with the ADMEM-based media. Based on the expression of pancreatic markers, in particular, *INS* and *SLC2A2*, we found that the N2B27-based media is more suitable for the induction of pancreatic *β*-like cells as compared to the ADMEM-based media.

### Morphological transition and pancreatic protein expression in the pancreatic β-like cells derived from BM-MSCs

**Figure 4:**
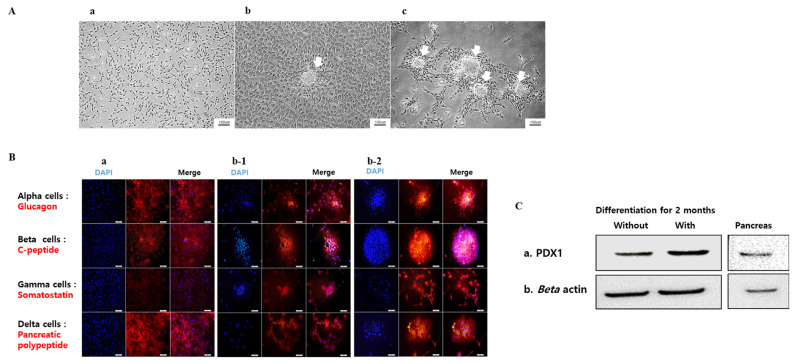
Morphological transition and pancreatic islets-specific protein expression analyses, after 1 month of cell differentiation. Morphological changes in (Aa) naïve GalTKO BM-MSCs, a control, (Ab) GalTKO BM-MSCs in ADMEM-based induction media after 5-azacytidine (5-Aza) treatment for 24 h, (Ac) GalTKO BM-MSCs in N2B27-based induction media after 5-Aza treatment for 24 h. Arrows represent islets-like clumps in the respective cells. (B) after 1 month of cell differentiation, immunofluorescence analysis of glucagon, C-peptide, somatostatin, and pancreatic polypeptide in GalTKO BM-MSCs induced with (a) ADMEM-based induction media, (b-1) N2B27-based induction media, and (b-2) N2B27-based induction media treated with 5-Aza for 24 h. The blue color represents nuclear staining (DAPI; 4′,6-diamidino-2-phenylindole) while red and green represent Alexa Fluor and fluorescein isothiocyanate (FITC) staining of secondary antibodies, respectively. Scale bar = 50 μm. (C) Western blot analysis of PDX1, before/after two months of pancreatic *β*-like cells induction using N2B27-based induction media treated with 5-Aza, (Cb) internal control beta Actin. Pancreatic tissue was used as a positive control. GalTKO, α-1,3-galactosyltransferase knockout; BM-MSCs, bone marrow-derived mesenchymal stem cells; ADMEM, advanced Dulbecco’s modified Eagle’s medium.

After pancreatic induction for one month, the GalTKO BM-MSCs showed changes in cellular morphology. The cells induced with the ADMEM-based media (5-Aza) showed clumping of cells to the center while the other cells showed a flattened morphology compared to the control GalTKO BM-MSCs (Figure 4Aa, 4Ab). Moreover, we found higher cellular clumping and formation of *β*-like cell clusters in cells induced with the N2B27-based media (5-Aza) compared the other two groups (Figure 4Ac). This pattern of cellular morphological changes from GalTKO BM-MSCs to pancreatic β-like cells was highest in the cells induced with the N2B27-based media (5-Aza) followed by the cells induced with the ADMEM-based media (5-Aza), and the least in the control GalTKO BM-MSCs.

The expression of pancreas-islets-specific proteins i.e., glucagon, c-peptide, somatostatin, and pancreatic polypeptide was observed by immunofluorescence in the pancreatic *β*-like cells derived from the GalTKO BM-MSCs after pancreatic induction for one month. Three samples: i) cells induced with the ADMEM-based media, ii) cells induced with the N2B27-based media, and iii) cells induced with the N2B27-based media with 5-Aza were selected for analyses. We found higher expression of glucagon, c-peptide, somatostatin, and PP in cells induced with the N2B27-based media (5-Aza) as compared with the other two groups (Figure 4B). The expression of these proteins was the highest in the cells induced with the N2B27-based media with 5-Aza followed by the cells induced with the N2B27-based media and the least in the cells induced with the ADMEM-based media. Estimation of protein levels by western blot revealed an increase in the expression of PDX1 in the 5-Aza group induced with the N2B27-based media for two months (Figure 4C).

### OCT4 promoter region methylation pattern

**Figure 5:**
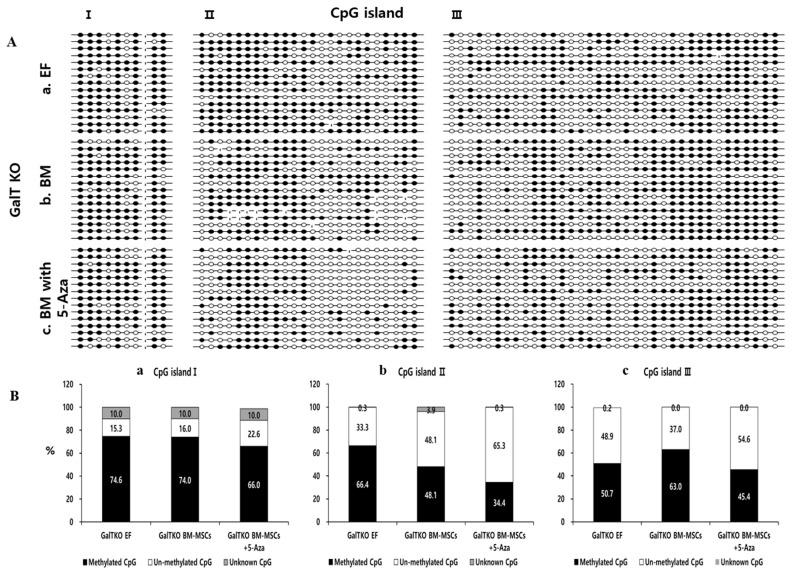
Epigenetic modification in the *OCT4* promoter region of GalTKO BM-MSCs before/after treatment with 5-azacytidine (5-Aza) for 24 h. (A) Representative image of three CpG islands in (Aa) Ear fibroblasts, (Ab) GalTKO BM-MSCs, and (Ac) GalTKO BM-MSC treated with 5-Aza for 24 h. ● represents methylated, ○ represents un-methylated, and - represents unknown CpGs. (B) Percentage of methylated and un-methylated CpGs present in CpG Island I (Ba), II (Bb), and III (Bc). *OCT4*, POU class 5 homeobox 1; GalTKO, α-1,3-galactosyltransferase knockout; BM-MSCs, bone marrow-derived mesenchymal stem cells.

In order to analyze the pluripotency of GalTKO BM-MSCs after 5-Aza treatment, we analyzed the promoter region of *OCT4* in GalT KO EFs, GalTKO BM-MSCs, and GalTKO BM-MSCs treated with 5-Aza (BM-MSCs 5-Aza). For this purpose, we checked the methylation pattern of 3 CpGs islands present in the *OCT4* promoter region using the bisulfite sequencing strategy (Figure 5A). In the first CpG island, the percentage of methylated CpG in the EF (74.6%) and GalTKO BM-MSCs (74%) were higher than that in the GalKO BM-MSCs + 5-Aza (66%) group. Furthermore, the un-methylated CpG percentage was also marginally higher in the GalKO BM-MSCs + 5-Aza group (22.6%), while 10% of the CpGs were marked as unknown in all three groups (Figure 5Ba). Similarly, upon analysis of the second CpG island, we found a decline in the number of methylated CpGs from the EF (66.38%) to GalKO BM-MSCs + 5-Aza (34.44%), while a drastic increase in the number of un-methylated CpGs in the GalKO BM-MSCs + 5-Aza (65.27%) as compared with the EF (33.33%) and GalTKO BM-MSCs (48.05%) (Figure 5Bb) groups was observed. A similar pattern of methylated (EF, 50.74%; GalTKO BM-MSCs, 62.96%; GalKO BM-MSCs + 5-Aza, 45.37%) and un-methylated (EF, 48.88%; GalTKO BM-MSCs, 37.03%; GalKO BM-MSCs + 5-Aza, 54.62%) CpGs were found in the third CpG island present in the promoter region of *OCT4* (Figure 5Bc). In all the three CpG islands we found a higher number of un-methylated CpGs in the GalKO BM-MSCs + 5-Aza as compared to the other two groups.

## DISCUSSION

BM-MSCs are considered as one of the most suitable sources of MSCs mainly because of the ease and accessibility of MSCs through aspiration procedures compared to that of the other sources [22]. Their multi-lineage potential makes these cells the most promising candidates for use in future regenerative medicines. However, a proper microenvironment is essential to transdifferentiate the BM-MSCs into the desired cells. In this study, we tried to enhance the differentiation ability of porcine GalTKO BM-MSCs into pancreatic *β*-like clumps by mimicking the cellular microenvironment using small molecules and epigenetic modulating factors.

DNA methylation plays an important role during somatic cell reprogramming and early embryonic development [19]. Furthermore, short exposure to a demethylation agent is sufficient to allow the direct conversion of adult mature cells into other lineages when induced with lineage-specific microenvironments [7]. 5-Aza has long been known to induce gene expression alterations and cellular phenotype transformations [20]. VPA is a major histone deacetylase inhibitor that promotes histone acetylation, accompanied by chromatin remodeling and affects DNA methylation [15]. Short exposure to 5-Aza leads to increased expression of pluripotent markers [7]. This is demonstrated in our results where a pre-exposure to 5-Aza and/or VPA, enhanced the expression of pluripotent markers in GalTKO BM-MSCs. This increase in the expression of pluripotent markers of GalTKO BM-MSCs supports the fact that 5-Aza works as a DNA methyltransferase inhibitor [23], activates the expression of silent genes, and alters the differentiation of MSCs [20]. Furthermore, we induced the GalTKO BM-MSCs pre-exposed to 5-Aza and/or VPA into pancreatic *β*-like cells using a step-wise ADMEM-based induction protocol. RT-qPCR analyses revealed that the 5-Aza treated GalTKO BM-MSCs showed the highest increase in the expression of *NEUROD1* which is involved in *β*-cell maturation from 15 weeks of gestation in humans [24]. However, these cells failed to achieve a pancreatic cell-fate as there was no dramatic change in *INS* levels. It was shown that GalTKO BM-MSCs treated with N2B27-chemically defined medium supplemented with growth factors could selectively induce the cells from embryonic stem cells (ESCs) to the endoderm/pancreas lineage [25]. Our results show that treatment with the simple N2B27-based media regardless of supplementation with epigenetic modifiers is sufficient to bring about significant epigenetic changes resulting in the by upregulation of both *INS* and *SLC2A2* and leading to the transdifferentiation of GalTKO BM-MSCs into mature pancreatic *β*-like cells. N2B27-based media supplemented with 5-Aza induced the upregulation of *NKX6* which is defined as “a key postnatal *β*-cell identity factor” [24] and enhanced the expression of INS. As the same epigenetic modifier produced different effects in cells growing in different culture media, we speculate that the mode of action of the epigenetic modifier is affected by the culture environment. Morphological observations also showed that the GalTKO BM-MSCs treated with the N2B27-based media supplemented with 5-Aza formed a higher number of cellular aggregates and islets-like clusters than other groups. The different RT-qPCR analysis demonstrated the expression of *INS* in the new pancreatic *β*-like cells; albeit, researchers previously claimed that the presence of insulin in these differentiated cells does not indicate intrinsic insulin secretion, rather it might be the insulin absorbed from the utilized culture media and sequestrated in these cells [26]. We re-affirmed INS expression by immunofluorescence of c-peptide, glucagon, somatostatin, and polypeptide in the pancreatic *β*-like cells suggesting that proinsulin synthesis was occurring in these cells and the INS expression observed was not from the insulin in the culture media.

DNA methylation of the *OCT4* gene regulatory region leads to *OCT4* silencing during embryonic development and differentiation of ESCs in human and mouse [27]. The analysis of porcine *OCT4* gene revealed the presence of three CpG islands upstream of the transcription start site [21]. In this study, we found comparatively higher demethylation in the GalTKO BM-MSCs compared to the EFs known to be hypermethylated [28]. Furthermore, the methylation pattern of three CpG islands present in the promoter region of *OCT4* revealed consistent demethylation in the 5-Aza treated GalTKO MB-MSCs demonstrating that 5-Aza enhanced the process of demethylation. In particular, among the three CpG islands, the number of hypo-methylated CpGs in second CpG island was much lower in the 5-Aza treated GalTKO BM-MSCs compared to that in the EFs and GalTKO BM-MSCs suggesting that this region is more important in epigenetic reprogramming. This demethylation of the *OCT4* promoter region after 5-Aza treatment supports the relatively higher expression level of *OCT4* in GalTKO BM-MSCs, suggesting activated stem cell capacity and the silence of tissue-specific genes. Moreover, analyzing all three CpG islands present in the porcine *OCT4* promoter region makes this study more impactful than previous studies where partial CpG islands were analyzed [29].

Taken together, we generated pancreatic *β*-like cells from GalTKO pig-derived BM-MSCs through microenvironment mimicking using an N2B27-based media and 5-Aza and revealed that for the transdifferentiation of BM-MSCs, a suitable induction media is more important than treatment with epigenetic modification agents. Furthermore, the methylation pattern of the second CpG island present in the *OCT4* promoter region further substantiated the effectiveness of 5-Aza. To our knowledge, this is the first study to employ the GalTKO BM-MSCs for the generation of pancreatic *β*-like cells by treating them with a DNA demethylation agent (5-Aza) and to analyze the methylation pattern of three CpG islands present in the promoter region of the porcine *OCT4* gene. As the use of human stem cells is limited due to ethical and immunological barriers; therefore, isolating stem cells from animals like GalTKO pig can provide an alternative to replace the human stem cells in future stem cells therapies. Furthermore, this study will provide an insight into the use of GalTKO BM-MSCs for the treatment of hyperglycemic disorders without the risk of hyperacute or other immunological rejections. The *in vivo* ability of the pancreatic *β*-like cells generated in this study to control blood sugar levels in diabetic animal model will be evaluated in future. Furthermore, as the epigenetic mechanisms (DNA methylations) plays important roles in cell-fate determination during pancreatic development; therefore, we will analyze the methylation pattern of other pluripotent as well as pancreatic genes in our future studies.

## Figures and Tables

**Table 1:** Primers used for real time polymerase chain reaction

Name		Sequence	Annealing Tm	Accession number
*GAPDH*	F	CCATCTTCCAGGAGCGAGAT	60	NM_001206359
	R	GCCTTCTCCATGGTCGTG AA		
*OCT4*	F	CGACCATCTGCCGTTTTGA	60	NM_001113060.1
	R	GCCGCAGCTTACACATGTTCT		
*SOX2*	F	GCGGCAACCAGAAGAACAG	60	NM_001123197.1
	R	CCACACCATGAAAGCGTTCA		
*NANOG*	F	CCCAGCTCCAGTTTCAGCAA	60	NM_001129971
	R	TCCCCAGCAGTTTCCAAGAC		
*INS*	F	GCAGAAGCGTGGCATCGT	60	NM_001109772.1
	R	GGCGGCCTAGTTGCAGTAGT		
*NEUROD1*	F	CCCGCCGCTCAGCAT	60	XM_021075510.1
	R	CGGACGGTTCGTGTTTGAA		
*GCG*	F	GAGACATGCTGAAGGGACCTTT	60	NM_214324.1
	R	GCAGCTTGGCCTTCCAAATA		
*SLC2A2*	F	CCCATCCCCTGGTTCATG	60	NM_001097417.1
	R	CAGGGCGTGGTCCTTGACT		
*NKX6*-*1*	F	TCGGGCCAGCAGATCTTC	60	XM_003129346.2
	R	CCCGCCAAGTATTTCGTTTG		
*PDX1*	F	GGTCTCAGGGCAGCGAAAA	60	NM_001141984.1
	R	CTCTGTGATGAGTTTCCGAGGAT		

*GAPDH*, glyceraldehyde-3-phosphate dehydrogenase; *OCT4*, POU class 5 homeobox 1; *SOX2*, SRY (sex determining region Y)-box 2; *NANOG*, Nanog homeobox; *INS*, insulin; *NEUROD1*, neurogenic differentiation 1; *GCG*, glucagon; *SLC2A2*, solute carrier family 2 (facilitated glucose transporter), member 2; *NKX6-1*, NK6 homeobox 1 *(NKX6); PDX1*, pancreatic and duodenal homeobox 1.
